# α-Arbutin ameliorates UVA-induced photoaging through regulation of the SIRT3/PGC-1α pathway

**DOI:** 10.3389/fphar.2024.1413530

**Published:** 2024-09-23

**Authors:** Fang Lu, Qi Zhou, Mengdi Liang, Huicong Liang, Yiwei Yu, Yang Li, Yan Zhang, Ling Lu, Yan Zheng, Jiejie Hao, Peng Shu, Jiankang Liu

**Affiliations:** ^1^ Key Laboratory of Marine Drugs, Ministry of Education, School of Medicine and Pharmacy, Ocean University of China, Qingdao, China; ^2^ HBN Research Institute and Biological Laboratory, Shenzhen Hujia Technology Co., Ltd., Shenzhen, Guangdong, China; ^3^ Department of Dermatology, The First Affiliated Hospital of Xi’an Jiaotong University, Xi’an, China; ^4^ State Key Laboratory Basis of Xinjiang Indigenous Medicinal Plants Resource Utilization, Xinjiang Technical Institute of Physics and Chemistry, Chinese Academy of Sciences, Urumqi, Xinjiang, China; ^5^ University of Chinese Academy of Sciences, Beijing, China; ^6^ School of Health and Life Sciences, University of Health and Rehabilitation Sciences, Qingdao, Shandong, China

**Keywords:** skin, photoprotective, HaCaT, sirtuin 3, mitochondrial biogenesis

## Abstract

Owing to its tyrosinase inhibitory activity, α-arbutin has been added to several skin care products as a skin-lightening agent. However, the protective effect of α-arbutin against ultraviolet A (UVA)-induced photoaging has not been well investigated. The present study was designed to investigate the photoprotective effect and mechanism of α-arbutin against UVA-induced photoaging. In vitro experiments, HaCaT cells were treated with UVA at a dose of 3 J/cm^2^ to evaluate the anti-photoaging effect of α-arbutin. α-Arbutin was found to exhibit a strong antioxidant effect by increasing glutathione (GSH) level and inhibiting reactive oxygen species (ROS) production. Meanwhile, α-arbutin markedly improved the expression of sirtuin 3 (SIRT3) and peroxisome proliferator-activated receptor γ coactivator 1 α (PGC-1α) proteins, initiating downstream signaling to increase mitochondrial membrane potential and mediate mitochondrial biogenesis, and improve mitochondrial structure significantly. In vivo analysis, the mice with shaved back hair were irradiated with a cumulative UVA dose of 10 J/cm^2^ and a cumulative ultraviolet B (UVB) dose of 0.63 J/cm^2^. The animal experiments demonstrated that α-arbutin increased the expression of SIRT3 and PGC-1α proteins in the back skin of mice, thereby reducing UV-induced skin damage. In conclusion, α-arbutin protects HaCaT cells and mice from UVA damage by regulating SIRT3/PGC-1α signaling pathway.

## 1 Introduction

The skin is the largest organ of the human body, accounting for approximately 10%–15% of the body weight (Jablonska-Trypuc et al., 2018). It is the first barrier between the body and the external environment, playing important roles in protection, regulation of temperature, sensation, secretion, excretion, and immunity (Gu et al., 2020). Epidemiological investigation showed that approximately 80% of exogenous skin aging is related to ultraviolet (UV) radiation, also termed skin photoaging (Zhang et al., 2023). UV radiation is generally classified into UVA (320–400 nm), UVB (280–320 nm), and UVC (190–280 nm) based on the wavelength range (Young et al., 2017; Egambaram et al., 2020). Solar UVC rays are absorbed by the ozone layer and do not reach the earth surface; thus, they do not pose a threat to human life. In contrast, UVA and UVB rays can penetrate the ozone layer, leading to skin damage (Xuan et al., 2019). A major fraction of UVB light is absorbed by macromolecular components of the skin, such as DNA, lipids, and aromatic amino acids of proteins (Schuch et al., 2017). UVB is the major cause of direct DNA damage and induces inflammation and immunosuppression, while UVA may play a greater role in skin photoaging, since it can penetrate the epidermis and reach deep into the dermis, thereby inducing connective tissue damage (Davies et al., 2017; Tang et al., 2024). Moreover, UVA can indirectly damage DNA by generating reactive oxygen species (ROS). This process progressively promotes oxidative stress, followed by the decrease of local antioxidant defenses as a key factor in the photoaging process (Swiader et al., 2021).

Mitochondria are the major source of ROS in UV-irradiated skin cells (Gomes et al., 2020). Numerous studies have linked sirtuin function to both arms of ROS: the regulation of ROS-mediated signaling, as well as the detoxification of damaging ROS(van de Ven et al., 2017). Investigations have shown that the changes in the expression of all classes of HDACs and SIRTs following UV irradiation and found that the expression of SIRT3 were significantly reduced by UV irradiation (Lee et al., 2021). Sirtuin3 (SIRT3) is considered the most important mitochondrial deacetylase, which plays an important role in regulating mitochondrial metabolism and energy production and maintaining redox homeostasis (Yang et al., 2016; Gomes et al., 2020). In addition, SIRT3 controls ROS levels in a more indirect way by promoting ETC function. It has been established that SIRT3 deacetylates multiple ETC proteins, such as NDUFA9 (complex I), SDHA (complex II) and so on to enhance energy production and improve ETC efficiency to reduce ROS production (Kumar and Lombard, 2015). The substrates of SIRT3 are very diverse and include enzymes, which serve unique and critical functions regulating metabolism, cell survival and longevity. SIRT3 can also cause activation of peroxisome proliferator-activated receptor α (PGC-1α), which activates Sirt3 gene promoter, leading to increased synthesis of Sirt3 mRNA transcripts (Pillai et al., 2015; Guan et al., 2021). The process of mitochondrial biogenesis is accompanied by mitochondrial DNA (mtDNA) replication and synthesis of mitochondrial proteins, leading to increased mitochondrial number and mass (Uoselis et al., 2023). PGC-1α is a key mitochondrial biogenesis stimulator, its downstream factors include estrogen-related receptor α (ERRα), NRF1, NRF2 and TFAM, which are responsible for replication, transcription, and translation of mtDNA (Park et al., 2017; Guan et al., 2021). TFAM is an essential protein that binds to mtDNA, regulates mitochondrial transcription initiation, and participates in mitochondrial genome replication (Kozhukhar and Alexeyev, 2019). Decrease in PGC-1α expression could give rise to mitochondrial dysfunction and aging (Park et al., 2017). However, SIRT3 KD impairs PGC-1α-induced mitochondrial biogenesis and blocks PGC-1α-induced mitochondria-related gene expression (Pillai et al., 2015). Therefore, increasing the mitochondrial biogenesis capacity of skin cells could be a valuable strategy for preventing UVA-induced oxidative stress and skin damage.

Recent years, skin health has also been more threatened due to the rising of exposures to UV radiation and chemical pollutants (Bais et al., 2020). Together with the growing global attention for environment sustainability, it has prompted the research of natural and eco-friendly bioactive compounds to improve human skin health without impact on the environment (Blunt et al., 2018). Arbutin (p-hydroxyphenyl-β-D-glucopyranoside) is a hydroquinone glycoside with two isomers including α-arbutin (4-hydroxyphenyl-α-glucopyranoside) and β-arbutin (4-hydroxyphenyl-α-glucopyranoside). Studies have shown that α-arbutin is 10 times more effective than natural arbutin (Saeedi et al., 2021). α-Arbutin is widely used in pharmaceutical and cosmetic industries owing to its well-known skin-lightening property as well as anti-oxidant, anti-microbial, and anti-inflammatory activities (Hazman et al., 2021). It has reported the antioxidant capacity *in vitro* of arbutin for free radical scavenging and reducing iron (Wang et al., 2020). Therefore, the use of α-arbutin as a safe and effective skin-whitening agent has received increasing attention in recent years (Saeedi et al., 2021). However, no study has comprehensively elaborated the antioxidant effects of α-arbutin against UVA irradiation on HaCaT cells. The main objective of this study was to determine the protective effect and mechanism of α-arbutin against UVA-induced photoaging both *in vitro* and *in vivo*.

## 2 Materials and methods

### 2.1 Materials and reagents

**FIGURE 1:**
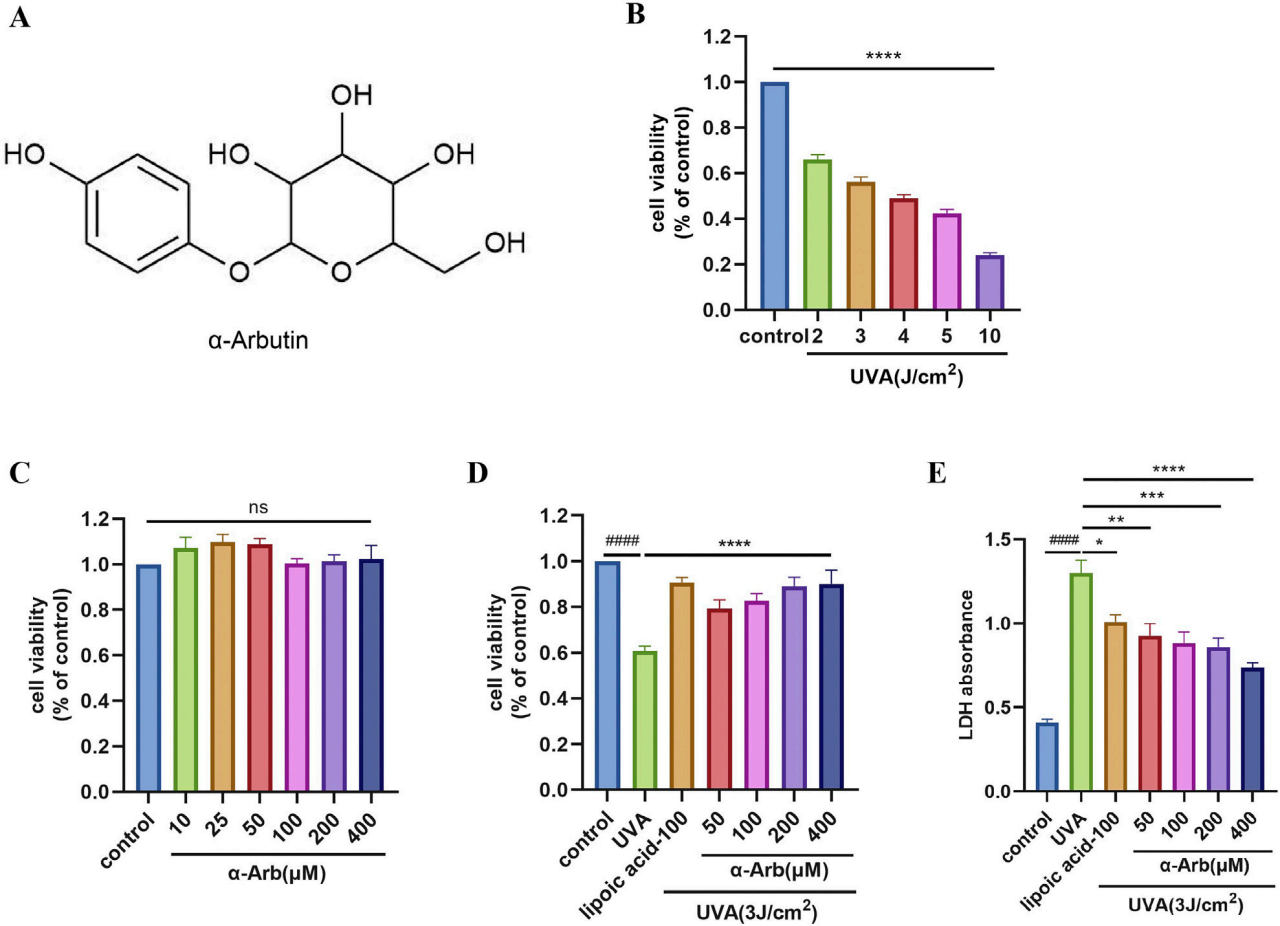
Effect of α-arbutin on the viability and LDH of HaCaT cells following UVA exposure. **(A)** Chemical structure of α-arbutin. **(B)** HaCaT cell viability was evaluated using the CCK-8 assay after irradiation of UVA. **(C)** HaCaT cell viability was evaluated using the CCK-8 assay after incubation with different concentrations of α-arbutin for 24 h **(D)** HaCaT cells were pretreated with different concentrations of α-arbutin (50 μM, 100 μM, 200 μM, 400 μM) for 24 h, and exposed to UVA (3 J/cm^2^). **(E)** LDH release. Values are presented as the mean ± SEM (n ≥ 3). ####*p* < 0.0001 vs. control group; *P < 0.05, **P < 0.01, ***p < 0.001, ****P < 0.0001 vs. UVA group.

The α-arbutin Figure 1A used in this study was provided by HBN Research Institute and Biological Laboratory (Shenzhen, China). Dulbecco’s modified Eagle’s medium, fetal bovine serum, and penicillin/streptomycin were purchased from Gibco (Grand Island, NY, United States). Cell Counting Kit-8 (CCK-8) assay, lactate dehydrogenase (LDH) assay, ROS assay, JC-1, Mito-Tracker green, and BCA protein assay kits Annexin V-FITC Apoptosis Detection kit were purchased from Beyotime Biotechnology (Shanghai, China). GSH assay and total antioxidant capacity (T-AOC) assay kits were purchased from Nanjing Jiancheng Bioengineering Institute (Nanjing, China). Mitochondrial respiratory chain complex I/NADH-CoQ and Ⅱ/succinic acid-Coenzyme Q reductase activity detection kits were purchased from Beijing Solarbio Science & Technology Co., Ltd. (Beijing, China). RNAFit and siRNA were purchased from HANBI Biotechnology Co., Ltd. Antibodies against Ki-67 (#11882), SIRT3 (#2627), PGC-1α (#2178), NDUFS1 (#70264), and SDHA (#11998) were purchased from Cell Signaling Technology (CST; Beverly, MA, United States). The antibody against β-actin (GB11001) and GAPDH (GB15004) was purchased from Sewell Biotechnology Co., Ltd. (Wuhan, China).

### 2.2 Cell culture and UVA treatments

The immortalized human skin keratinocyte cell line HaCaT was obtained from the Chinese Academy of Sciences Cell Bank (Shanghai, China). HaCaT cells were cultured in DMEM supplemented with 10% heat-inactivated FBS and 1% penicillin/streptomycin at 37°C in an atmosphere composed of 95% air and 5% CO_2_. All experiments were performed using cells between the third and seventh passage.

Initially, HaCaT cells were seeded in a 96-well microplate and incubated at 37°C in a humidified atmosphere for 12 h. Thereafter, the cells were washed with PBS and resuspended in fresh phenol red-free DMEM containing 10% FBS, and the cells were exposed to different doses of UVA irradiation after the lid of cell culture plate was removed. Subsequently, the cells were moved to a cell incubator and incubated for 12 h after UVA irradiation.

### 2.3 CCK-8 assay

HaCaT cells were seeded in 96-well plates at a density of 1 × 10^4^ cells/100 μL and cultured for 24 h. Next, the cells were treated with the test drug for 24 h. After incubation, the cells were resuspended in fresh phenol red-free DMEM containing the test drug. The cells were divided into the following groups: control; UVA alone; and UVA + α-arbutin (50, 100, 200, 400 μM). Subsequently, the cells were irradiated according to the grouping using a UVA lamp. And HaCaT cells were cultured for another 12 h in medium containing different concentrations of α-arbutin. The protective effects of α-arbutin on cells were determined by the CCK-8 assay. The absorbance at 490 nm wavelength was measured directly with a microplate reader.

### 2.4 Evaluation of biochemical indices

The cell supernatants and proteins were collected and analyzed according to the instructions provided by the manufacturer. Following treatment, LDH levels in cell supernatants were quantified using a microplate reader. Thereafter, intracellular levels of GSH and T-AOC were measured according to the corresponding instructions provided by the manufacturer.

### 2.5 Detection of intracellular ROS

The production of intracellular ROS following UVA radiation was determined with a microplate reader and fluorescence microscopy using DCFH-DA. Following pretreatment with α-arbutin and UVA irradiation, the cells were washed with PBS, incubated with 10 μM DCFH-DA in DMEM at 37°C for 20 min, and washed again with PBS. Finally, the fluorescence was measured at excitation wavelength 488 nm and emission wavelength 535 nm using a spectrophotometer. Cells cultured in confocal laser culture dish were subjected to the process described above and examined using a confocal laser scanning microscope.

### 2.6 Immunofluorescence

HaCaT cells were seeded in confocal glass bottom dishes and incubated for an additional 12 h after receiving UVA irradiation. Cells were washed once with and fixed with 4% paraformaldehyde for 15 min. Subsequently, they were washed 3 times with PBS (5 min per wash) and permeabilized with 0.1% TritonX-100 in PBS for 5 min. After washing thrice, the cells were blocked with blocking buffer for 1 h at room temperature, and incubated with primary antibodies in a moist chamber at 4°C overnight. Cells were washed 3 times with PBS and incubated with FITC-labeled secondary antibody for 1 h at 37°C. After washing thrice with PBS, DAPI staining solution was added to stain the nuclei, and the cells were incubated at 37°C in the dark for 15 min. After washing three times with PBS, the cells were observed under a laser confocal microscope.

### 2.7 Annexin V/PI staining and flow cytometry

Apoptosis was analyzed by flow cytometry using Annexin-V fluorescein isothiocyanate (FITC) and propidium iodide (PI) detection kit (Beyotime, China). HaCaT cells were seeded in 6-well plates and incubated for an additional 12 h after receiving UVA irradiation. Then, cells were collected and incubated with a mixture containing Annexin V-FITC and PI for 20 min in the dark and detected on a BD flow cytometer (BD Biosciences, San Jose, CA). The data were further analyzed by using FlowJo software.

### 2.8 Measurement of MMP (ΔΨm)

Determination of MMP (ΔΨm) was carried out using the proportion dye JC-1, which is a dual emission potential-sensitive probe. The cells were cultured in 6-well plates and incubated with JC-1 staining solution for 20 min at 37°C, washed twice with PBS. The fluorescence of JC-1 monomers was measured at excitation wavelength 490 nm and emission wavelength 530 nm, and the fluorescence of JC-1 aggregates was measured at excitation wavelength 525 nm and emission wavelength 590 nm using a spectrophotometer. The ΔΨm was calculated using the following formula: ΔΨm = JC-1 aggregates/JC-1 monomers.

### 2.9 Determination of mitochondrial content

The mitochondrial content following UVA radiation was determined by a microplate reader using Mito-Tracker Green. HaCaT cells were seeded in 6-well plates, Mito-Tracker Green was added to a final concentration of 100 nm in the dark, and the cells were cultured for 30 min at 37°C. After washing thrice with PBS, the fluorescence for each group was detected at excitation wavelength 490 nm and emission wave-length 516 nm with a microplate reader.

### 2.10 Transmission electron microscopy (TEM) analysis of mitochondria

HaCaT cells were seeded in 6-well plates at a density of 4 × 10^5^/well, pretreated with α-arbutin for 24 h, and incubated for 12 h after UVA irradiation. Finally, HaCaT cells were collected and fixed in glutaraldehyde at 4°C overnight. Thin sample sections were produced. TEM (JEM-100CX II) was utilized to assess the morphological changes in mitochondria.

### 2.11 Molecular docking

All protein structure files of SIRT3 were downloaded from RCSB PDB (https://www.rcsb.org/). The protein structures were prepared by DockPrep module and minimized with the ff14SB/gaff force field in UCSF Chimera (http://www.cgl.ucsf.edu/chimera/). Finally, all the structures were converted to pdbqt format with all hydrogens kept by rdkit2pdbqt.py script (https://github.com/biocheming/watvina, accessed on 26 December 2023, Ximing Xu, QingDao, China). The docking box size of each protein is set by extending ligand coordinate 5 Å on each dimension. Docking studies of SIRT3 structures and arbutin were performed using watvina (https://github.com/biocheming/watvina, accessed on 26 December 2023 Ximing Xu, QingDao, China), which was developed by our group. Based on the Autodock vina molecular docking engine, watvina is optimized on scoring function and conformation searching algorithm. The scoring function of watvina consists of Van der Waals, hydrogen bonds, polar-polar repulsion or hydrophobic attraction. Unlike Autodock vina, watvina considers the contribution of all hydrogen atoms. Conformation searching adopts a simplified genetic algorithm-simulated annealing-BFGS combination strategy; in addition, a torsion penalty is calculated for conjugated rotable single bonds.

### 2.12 Molecular dynamics simulation

Molecular dynamics (MD) simulation performed by the AMBER software (https://ambermd.org/) plays a significant role in inspecting ligand-binding interactions and stability. Molecular system was processed by ff19SB force field and gaff2 force field, solvated with crystallographic water (TIP4P-EW) molecules under rectangular periodic boundary conditions for a 12 Å buffer region, and neutralized by adding Na^+^ as counterions. The method of system minimization was shifted from steepest descent to conjugate gradient after 5,000 cycles, with a maximum of 10,000 cycles. After heating the system for 50 ps with Langevin dynamics and reaching 300 k, a 50 ps NVT ensemble was performed. Then a production MD was carried out by adding Berendsen barostat to control the pressure on 1 bar at 300 K for 100 ns. A time step of 0.002 ps was set, and energy and structures were recorded at every 100 ps. The result of the MD simulation was visualized and analyzed by the VMD software.

### 2.13 Surface plasmon resonance (SPR) studies

The SPR experiments were carried out using a Biacore T200 instrument (Biacore; GE Healthcare) at 25°C. The CM5 chip surface was activated after mixing 0.4 mol/L EDC and 0.1 mol/L NHS at a 1:1 ratio. Next, the chip surface was blocked with ethanolamine (pH 8.5). The analytes and different concentrations of α-arbutin diluted using PBSP buffer were injected and passed over the immobilized SIRT3 sensor surface. The flow rate was 30 μL/min, the binding time was 90 s, and the dissociation time was 90 s. The experimental data were analyzed using Biacore T200 evaluation software.

### 2.14 Western blotting analysis

HaCaT cells were seeded in 6-well plates at a density of 4 × 10^5^/well, pretreated with α-arbutin for 24 h, and incubated for 12 h after UVA irradiation. The cells were incubated with the western lysate on ice for 30 min. Next, the homogenate was centrifuged at 14,000 rpm for 5 min at 4°C to remove cell debris. The protein concentration was determined using the BCA Protein Assay Kit. Cell lysates (25 mg protein per lane) were subjected to 10% SDS-PAGE, transferred onto a nitrocellulose membrane, and blocked with 5% BSA/TBST for 2 h at room temperature. Membranes were incubated with primary antibodies against β-actin, SIRT3, PGC-1α, NDUFS1, and SDHA in 5% bovine serum albumin/TBST overnight at 4°C. After washing six times with TBST, the membrane was incubated with alkaline phosphatase-conjugated secondary antibody for 1 h at room temperature. Western blots were developed using BCIP/NBT alkaline phosphatase color development kit and quantified by scanning densitometry. The relative protein expression was determined through normalization to β-actin expression. Results are expressed as a percentage of the control. Finally, ImageJ software (National Institutes of Health, Bethesda, MD, United States) was used for grayscale densitometric analysis.

### 2.15 siRNA transfection

HaCaT cells at approximately 50% confluence were transfected using RNAFit according to manufacturer’s guidelines (HANBI Biotechnology Co., Ltd., Shanghai, China). Si-SIRT3-1, si-SIRT3-2, and si-SIRT3-3 were obtained from HANBI Biotechnology Co., LTD. HaCaT cells were transfected with 50 nM siRNA in DMEM for 48 h followed by treatment with or without 200 μM α-arbutin for 24 h. The sample were collected and stored at −80°C until assayed for proteins and mRNA expression. The sequences of si-SIRT3 genes are shown in Table 1.

**TABLE 1:** Genes sequences of si-SIRT3 are as follows.

Genes	Forward	Reverse
si-SIRT3-1	5′-GGU​GGA​AGA​AGG​UCC​AUA​UAT​T-3′	5′-AUA​UGG​ACC​UUC​UUC​CAC​CTT-3′
si-SIRT3-2	5′-GCA​GAA​CAU​CGA​UGG​GCU​UTT-3′	5′-AAG​CCC​AUC​GAU​GUU​CUG​CTT-3′
si-SIRT3-3	5′-GGA​AAG​CCU​AGU​GGA​GCU​UTT-3′	5′-AAG​CUC​CAC​UAG​GCU​UUC​CTT-3′

### 2.16 Quantitative Real-Time RT-PCR assay

HaCaT cells (4 × 10^5^cells/well) were treated with α-arbutin. After incubation for 24 h, the solution was discarded, and the cells were irradiated with UVA (3 J/cm^2^). After 12 h, HaCaT cells were washed twice with ice-cold PBS. Total RNA was isolated using SparkZol reagent, and RNA (2 µg) was reverse transcribed into cDNA using Hiscript III All-in-one RT SuperMix. Finally, target cDNA levels were quantified by RT-PCR using a QuantStudioTM 3 Real-Time PCR instrument. The quantitative PCR reactions were performed at 95°C for 3 min, followed by 40 amplification cycles (10 s at 95°C and 30 s at 60°C). Relative expression levels were determined through normalization to those of β-actin using the 2^−ΔΔCT^ method. The primers used are shown in Table 2.

**TABLE 2:** Primer sequences used for RT-PCR were as follows.

Genes	Forward	Reverse
SIRT3	5′-TGC​AGA​AGT​AGC​AGT​TCA​GTG-3′	5′-GCT​TCC​TCT​AGT​GAC​ACT​GTT​AG-3′
PGC-1α	5′-TTG​CTA​AAC​GAC​TCC​GAG​AAC​A-3′	5′-CAA​CTG​ACC​CAA​ACA​TCA​TAC​CC-3′
NRF1	5′-CGC​AGC​ACC​TTT​GGA​GAA-3′	5′-CCC​GAC​CTG​TGG​AAT​ACT​TG-3′
NRF2	5′-CAG​CGA​CCT​TCG​CAA​ACA​AC-3′	5′-CAT​GAT​GAG​CTG​TGG​ACC​GT-3′
TFAM	5′-GGC​ACA​GGA​AAC​CAG​TTA​GG-3′	5′-CAG​AAC​ACC​GTG​GCT​TCT​AC-3′
D-loop	5′-TCT​GTC​TTT​GAT​TCC​TGC​CTC​A-3′	5′-AGT​GGC​TGT​GCA​GAC​ATT​CA-3′
β-actin	5′-AGA​GCT​ACG​AGC​TGC​CTG​AC-3′	5′-AGC​ACT​GTG​TTG​GCG​TAC​AG-3′

### 2.17 Animal experiments

Female BALB/c mice (6–8 weeks) were obtained from Jinan Pengyue Laboratory Animal Co., Ltd. (Jinan, China). The mice were maintained at an ambient temperature of 25°C under a light/dark cycle of 12 h with food and water. Prior to the experiment, the back hair of the mice was shaved, and the mice were placed in the experimental environment for adaptive rearing for 1 week.

We selected three UVA lamps with a peak of 340 nm and two UVB lamps with a peak of 313 nm, which were arranged alternately to simulate solar radiation. The UV irradiance was measured with an ultraviolet irradiance meter (TS280E). The UV lamp was located approximately 40 cm above the mice. The mice were irradiated for 1 h daily and every other day for 15 days with a cumulative UVA dose of 10 J/cm^2^ and a cumulative UVB dose of 0.63 J/cm^2^ α-Arbutin containing hydrogel was applied to the back skin of the mice before irradiation. BALB/c mice were randomly divided into the following four groups: control group (no UV irradiation), model group (UV irradiation + solvent application), α-arbutin low dose group (UV irradiation +15 mg/kg/d α-arbutin), α-arbutin high dose group (UV irradiation +45 mg/kg/d α-arbutin). The experimental protocol was approved by the Institution Animal Ethics Committee of Ocean University of China (OUC-SMP-2023-0907). Efforts have been made in this study to minimize animal suffering and the number of animals used.

### 2.18 Histological analysis

On day 15, dorsal skin samples (approximately 0.5 × 0.5 cm) were obtained by quick stripping, dehydrated in ethanol, embedded in paraffin, and sectioned. The sections were stained with H&E and Masson’s trichrome stain. The histopathologic changes were examined under a light microscope. For each sample, three random fields were selected and photographed.

### 2.19 Immunohistochemical staining

The detection of SIRT3 and PGC-1α expression in skin samples obtained from mice was performed by immunohistochemistry. Briefly, skin sections were pretreated in a microwave oven to inactivate enzymes, blocked, and reacted with primary and secondary antibodies. The samples were chromogenized, counterstained, decolorized, sealed, and finally scanned with a digital pathology slide scanner.

### 2.20 Statistical analysis

Data are expressed as the mean ± SEM. GraphPad Prism 8.0.2 software (GraphPad Software Inc., San Diego, CA, United States) was used for statistical analysis, and one-way analysis of variance (ANOVA) was performed. *P*-values <0.05 denote statistical significance.

## 3 Results

### 3.1 α-Arbutin protects UVA-induced decrease of HaCaT cell viability

We selected HaCaT cells and treated them with UVA. We found that UVA at doses of 2, 3, 4, 5, and 10 J/cm^2^ reduced the viability of HaCaT cells. A dose of 3 J/cm^2^ of UVA (about 45% decrease of cell viability) was chosen for subsequent experiments (Figure 1B).

To assess the cytotoxicity of α-arbutin, HaCaT cells were treated with different concentrations of α-arbutin, and cell viability was evaluated using the CCK-8 assay. The results indicated that α-arbutin did not exert a toxic effect on HaCaT cells at concentrations rang of 400 μM (Figure 1C). α-Lipoic acid is a widely used antioxidant that protects mitochondria from oxidative damage *in vivo* (Salehi et al., 2019). Moreover, it has been shown that lipoic acid improves mitochondrial function in nonalcoholic steatosis through the stimulation of SIRT3 and PGC-1α(Valdecantos et al., 2012), thus we chose α-lipoic acid as a positive control. Moreover, pretreatment with 50, 100, 200, and 400 µM α-arbutin significantly increased the cell viability of HaCaT cells after exposure to UVA (Figure 1D). The results of LDH release assays corroborated these findings (Figure 1E).

### 3.2 α-Arbutin enhances UVA-induced antioxidant effect in HaCaT cells

**FIGURE 2:**
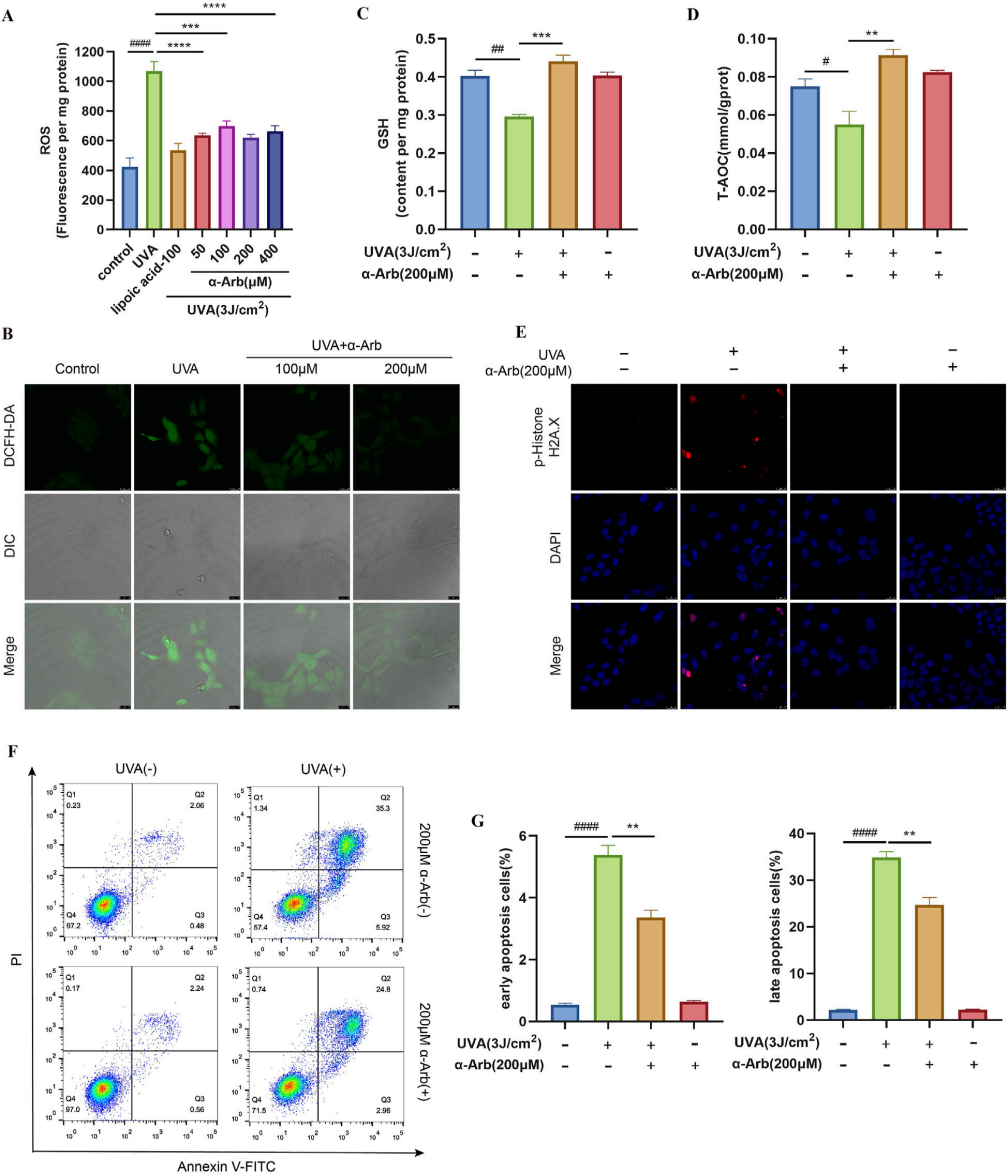
Effect of α-arbutin on antioxidant activity. **(A)** The cells were incubated with 10 μM DCFH-DA for the ROS production assay. **(B)** ROS levels was further observed by laser confocal microscopy (magnification: ×630; scale bar: 25 μM). **(C)** GSH and **(D)** T-AOC levels. **(E)** Phospho-Histone H2A.X was evaluated by immunofluorescence staining. **(F, G)** HaCaT cells were stained with Annexin V-FITC/PI and analyzed by using flow cytometry. Values are presented as the mean ± SEM (n ≥ 3). #*P* < 0.05, ##*P* < 0.01, ####*P* < 0.0001 vs. control group; **P < 0.01, ***P < 0.001, ****P < 0.0001 vs. UVA group.

Mitochondria are the source of ROS and also one of the main targets of ROS destruction, and severe mitochondrial oxidative damage usually leads to mitochondrial dysfunction (Guo et al., 2022). The production of ROS in HaCaT cells after treatment with UVA was detected using DCFH-DA. ROS production was significantly increased in HaCaT cells treated with UVA, and pretreatment with α-arbutin inhibited ROS levels (Figure 2A). The intensity of ROS green fluorescence was further observed by laser confocal microscopy. Cells exposed to UVA showed stronger green fluorescence compared with control cells. The green fluorescence was significantly attenuated in the α-arbutin group (Figure 2B), further confirming that α-arbutin significantly inhibited intracellular ROS production. Based on the above results, we selected the dose of 200 µM for subsequent studies.

GSH plays an important role in scavenging oxidant species and maintaining the redox homeostasis in cells. The deficiency of GSH can cause ROS accumulation and amplify intracellular oxidative stress, inducing cell dysfunction and death (Niu et al., 2021). The GSH production was examined, and the results are shown in Figure 2C. Treatment with UVA resulted in a significant decrease in GSH production in HaCaT cells. Notably, α-arbutin significantly inhibited the reduction of cellular GSH.

The levels of various endogenous antioxidants in the body can reflect the T-AOC of the body. The T-AOC of the UVA-irradiated cells was significantly reduced. However, the T-AOC was significantly increased after treatment with 200 µM α-arbutin (Figure 2D). The findings demonstrated that α-arbutin increased the antioxidant effect of HaCaT cells.

### 3.3 α-Arbutin protects HaCaT cells from UVA-induced DNA damage and cell apoptosis

DNA is one of the prime intranuclear targets in photosensitized stress (Liu et al., 2022). We demonstrated that exposure to UVA increased the proportion of phospho-Histone H2A.X (DNA damage marker)-positive cells and staining intensity, while pretreatment with α-arbutin markedly reduced the generation of phospho-Histone H2A.X (Figure 2E).

Annexin V/PI double staining showed that the ratio of apoptosis cells was increased in the UVA-irradiated group but reduced after α-arbutin treatment (Figures 2F, G). The results indicated that α-arbutin had an inhibitory effect on apoptosis induced by UVA.

### 3.4 α-Arbutin increases UVA-induced MMP and mitochondrial content in HaCaT cells

**FIGURE 3:**
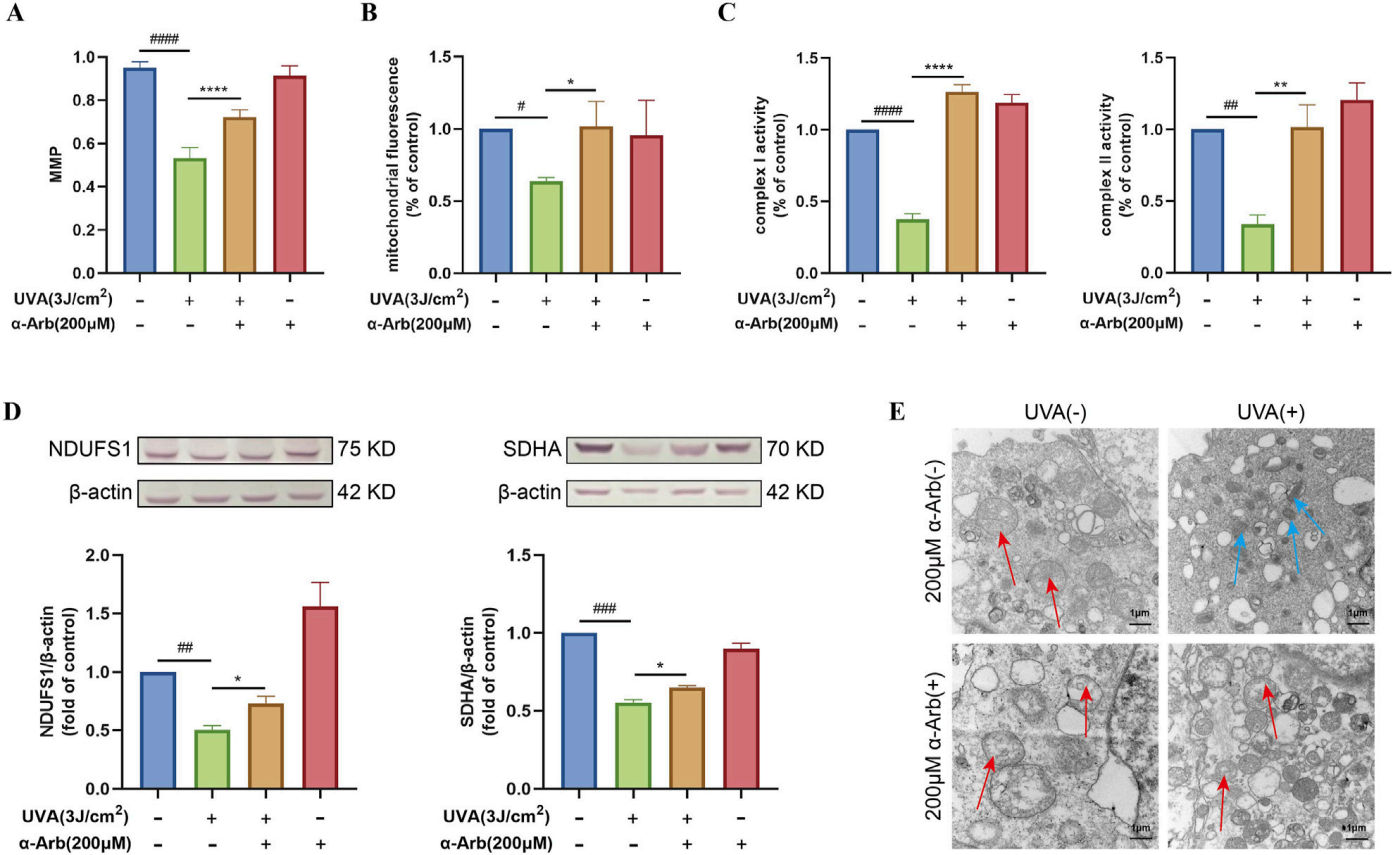
Effect of α-arbutin on the mitochondrial function of HaCaT cells after UVA exposure. **(A)** The MMP was measured. **(B)** The cells were incubated with 100 nM Mito-Tracker Green for the mitochondrial content assay. **(C)** Activities of complex I and II. **(D)** Western blotting analysis of NDUFS1 and SDHA protein expression. **(E)** TEM analysis of mitochondria. Values are presented as the mean ± SEM (n ≥ 3). #*P* < 0.05, ##*P* < 0.01, ###*P* < 0.001, ####*P* < 0.0001 vs. control group; *P < 0.05, **P < 0.01, ****P < 0.0001 vs. UVA group.

HaCaT cells were exposed to UVA, and the MMP of the HaCaT cells was measured using the JC-1 method. As shown in Figure 3A, UVA induced a significant decrease in MMP in HaCaT cells. However, pretreatment of HaCaT cells with α-arbutin significantly reversed the UVA-induced decrease in MMP. We stained HaCaT cells with Mito-Tracker Green and the change in mitochondrial fluorescence, indirectly reflected the mitochondrial content. The results showed that, after UVA irradiation, the intracellular fluorescence intensity was significantly decreased compared with that recorded in the control group. Pretreatment with 200 µM α-arbutin significantly inhibited the decrease in mitochondrial content caused by UVA damage (Figure 3B).

### 3.5 α-Arbutin enhances the activity and expression of mitochondrial complexes in UVA-induced HaCaT cells

Figure 3C showed that 200 μM of α-arbutin pretreatment significantly increased the activity of mitochondrial complex I and mitochondrial complex II in UVA-exposed HaCaT cells. Western blotting analysis was used to determine the expression of mitochondrial complex I (NDUFS1) and complex II (SDHA). The expression levels of NDUFS1 and SDHA proteins were significantly downregulated in UVA-irradiated HaCaT cells compared with control (Figure 3D). Nevertheless, upregulation of both proteins was observed after treatment with 200 µM α-arbutin.

### 3.6 α-Arbutin protects the mitochondrial structure in UVA-induced HaCaT cells under TEM analysis

The mitochondrial structure and shape of cristae determine mitochondrial respiratory efficiency (Jin et al., 2024). The morphological changes in mitochondria after different treatment were observed by TEM. As shown in Figure 3E, UVA-irradiated cells showed a disrupted mitochondrial structure, abnormally shaped mitochondria, and fragmented or destroyed mitochondrial cristae. Importantly, treatment with α-arbutin protected the mitochondrial structure. In addition, the mitochondria morphology was significantly improved.

### 3.7 Interaction between α-arbutin and SIRT3 protein

**FIGURE 4:**
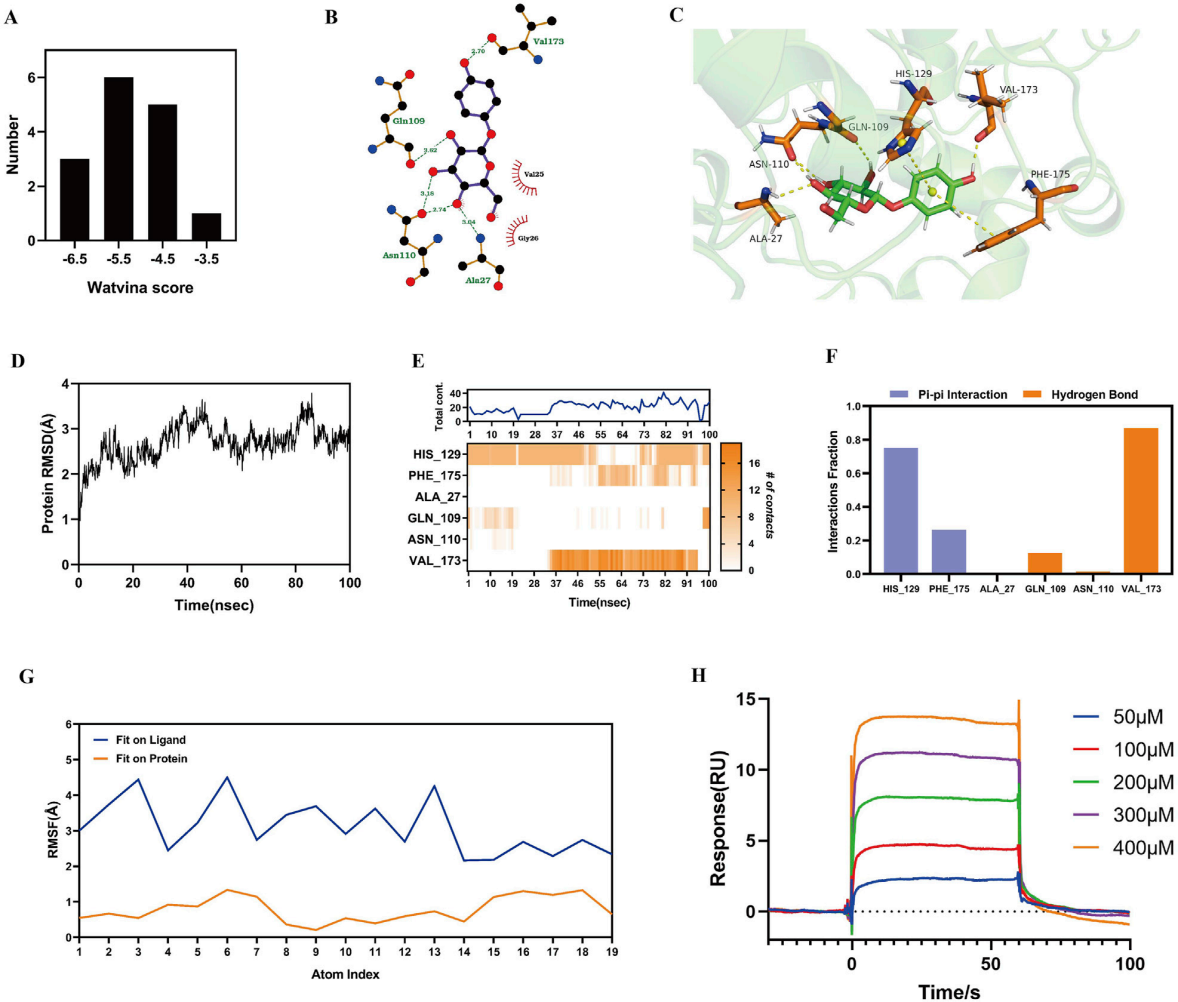
Interaction of α-arbutin with SIRT3. **(A)** The docking score distribution diagram of arbutin and 15 crystal structures of SIRT3. **(B)** The 2D interaction structures between arbutin and SIRT3. Hydrogen bonds are shown as green dotted lines, while the spoked arcs represent residues making nonbonded contacts with the ligand. **(C)** The 3D interaction structures between arbutin and SIRT3. **(D)** The Root Mean Square Deviation (RMSD) of SIRT3 backbone. **(E)** The interactions between key residues and SIRT3 during the whole simulation process. **(F)** The main interactions fraction between arbutin and SIRT3. **(G)** The Ligand Root Mean Square Fluctuation (L-RMSF) of arbutin in SIRT3. **(H)** Binding affinity between α-arbutin and SIRT3.

Molecular docking was performed to study the interactions between α-arbutin and SIRT3. Fifteen crystal structures of SIRT3 were obtained from Protein Data Bank (PDB), and the docking score of these fifteen crystal structures and arbutin was calculated by watvina respectively. The docking score distribution of arbutin and fifteen rystal structures of SIRT3 is shown in Figure 4A. The SIRT3 (PDBID:4JT8) had the best score with α-arbutin, with a score of −6.36, and the docking results are shown in Figures 4B, C. The binding stability of α-arbutin and SIRT3 (PDBID:4JT8) was further simulated by molecular dynamics (MD) simulations. The Root Mean Square Deviation (RMSD) of SIRT3 backbone in the complex was <4 Å, indicating that SIRT3 had a stable conformation during the simulation (Figure 4D). The Root Mean Square Fluctuation (RMSF) values of heavy atoms of arbutin were <2 Å relative to the protein, indicating a relatively firm binding to the protein pocket (Figure 4E). As shown in Figures 4F, G, HIS129, PHE175, GLN109 and VAL173 were key residues. The pi-pi and hydrogen bond interactions fraction value corresponding to HIS129 and VAL173 were both above 0.7, which were respectively 0.75 and 0.87, indicating that two residues and α-arbutin maintained constant interactions throughout the simulation time.

We used a SPR assay to test the binding affinity between α-arbutin and SIRT3 (Figure 4H). The data showed that α-arbutin bound SIRT3 in a dose-dependent manner. After fitting, the equilibrium dissociation constant (KD) value of α-arbutin and SIRT3 was calculated to be 167.8 μM, thereby confirming a certain binding effect.

### 3.8 α-Arbutin increases the expression of key proteins of the SIRT3/PGC-1α signaling pathway

**FIGURE 5:**
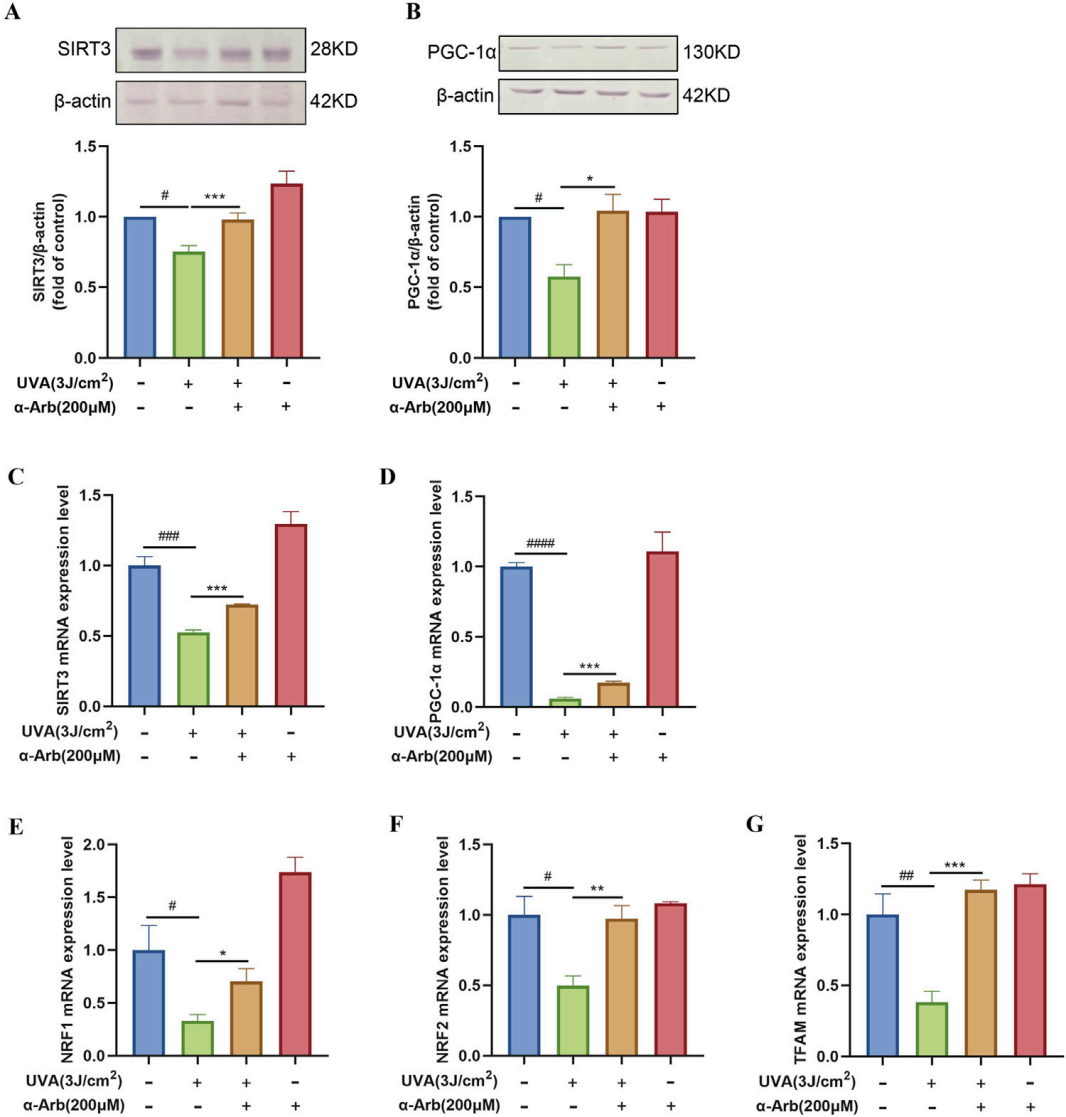
Effect of α-arbutin on the SIRT3/PGC-1α signaling pathway. **(A)** The expression levels of SIRT3 and **(B)** PGC-1α proteins. The mRNA expression levels of **(C)** SIRT3, **(D)** PGC-1α, **(E)** NRF1, **(F)** NRF2, and **(G)** TFAM were quantified by RT-PCR. Values are presented as the mean ± SEM (n ≥ 3). #*P* < 0.05, ##*P* < 0.01, ###*P* < 0.001, ####*P* < 0.0001 vs. control group; *P < 0.05, **P < 0.01, ***P < 0.001 vs. UVA group.

We performed Western blotting analysis to determine the protein expression of SIRT3 and PGC-1α. The expression levels of SIRT3 and PGC-1α proteins were significantly downregulated in UVA-irradiated HaCaT cells compared with control cells. After treatment with 200 μM α-arbutin, we observed an upregulation of SIRT3 and PGC-1α protein expression (Figures 5A, B).

### 3.9 α-Arbutin increases the mRNA expression of the SIRT3/PGC-1α signaling pathway

The role of α-arbutin in the SIRT3 signaling pathway was explored, and quantitative PCR assays were used to assess the mRNA expression levels of SIRT3. SIRT3 mRNA expression levels were significantly increased after treatment with α-arbutin compared with control (Figure 5C). We sought to further validate the role of α-arbutin in metabolism *in vitro* by investigating its effects on the SIRT3 pathway. Therefore, we determined the expression of target genes downstream of the SIRT3 pathway. The mRNA expression of PGC-1α was significantly increased after treatment with 200 μM α-arbutin. Moreover, α-arbutin significantly increased the levels of NRF1, NRF2, and TFAM (Figures 5D–G).

### 3.10 Effect of siSIRT3 on SIRT3 pathway

**FIGURE 6:**
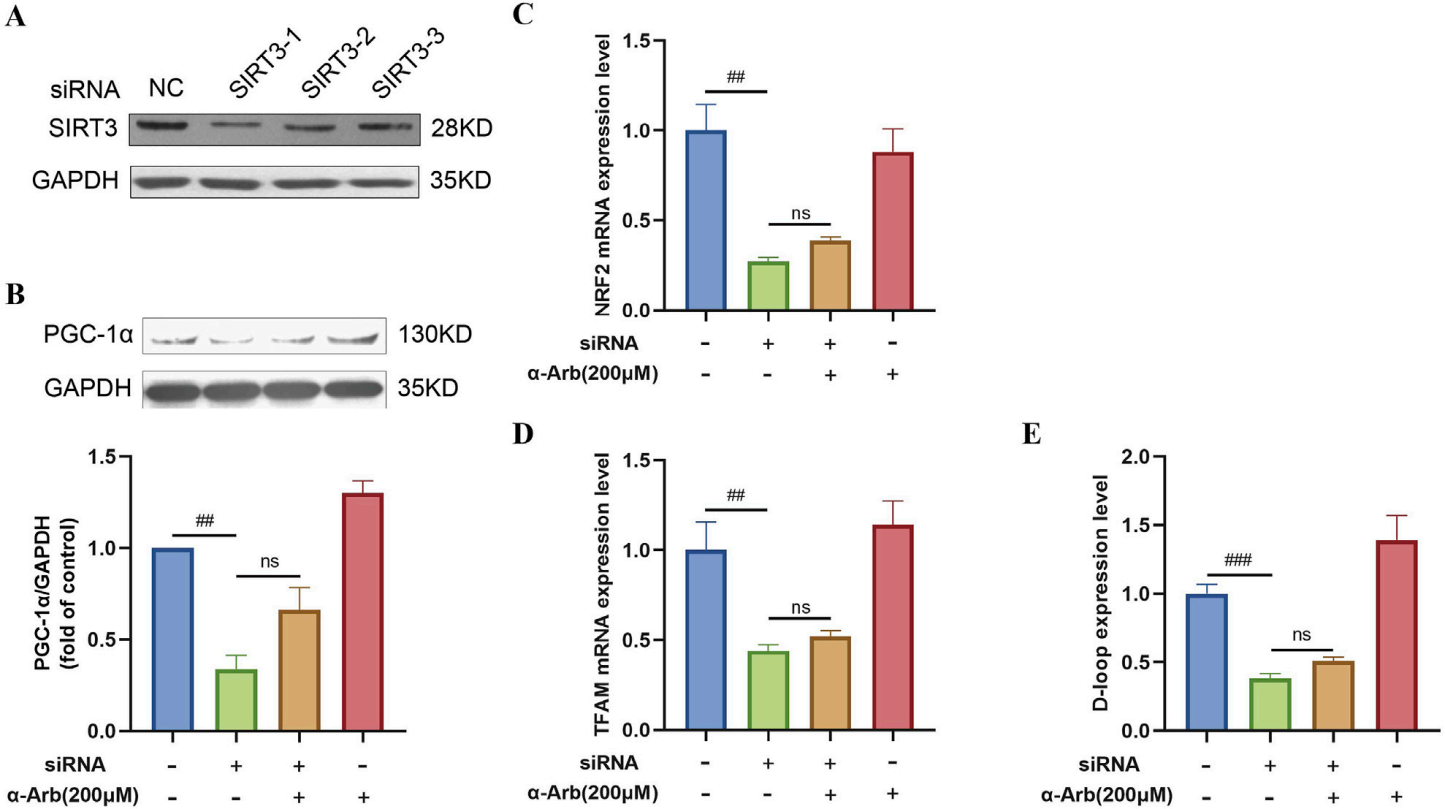
Effect of siSIRT3 on SIRT3 pathway. **(A)** siRNA inhibited the expression of the SIRT3 protein in HaCaT cells. **(B)** The addition of α-arbutin could not reverse the expression of PGC-1α protein. **(C–E)** The mRNA expression of NRF1, TFAM and D-loop expression after SIRT3 inhibition. Values are presented as the mean ± SEM (n = 3). #*P* < 0.05, ##*P* < 0.01, ###*P* < 0.001 vs. control group.

To test whether the protective effect of α-arbutin was SIRT3 protein dependent, we performed the following experiments. As shown in Figure 6A, siRNA3-1 was selected to transfected cells. In RNAfit transfected cells, the addition of α-arbutin could not significantly reverse the expression of the PGC-1α protein (Figure 6B). At the same time, α-arbutin could not reverse the decrease of the mRNA expression of NRF2, TFAM and the expression of D-loop (Figures 6C–E). Clearly, the protection of α-arbutin was dependent on the SIRT3 protein.

### 3.11 α-Arbutin improves the macroscopic appearance and histological changes in photoaged mice skin

**FIGURE 7:**
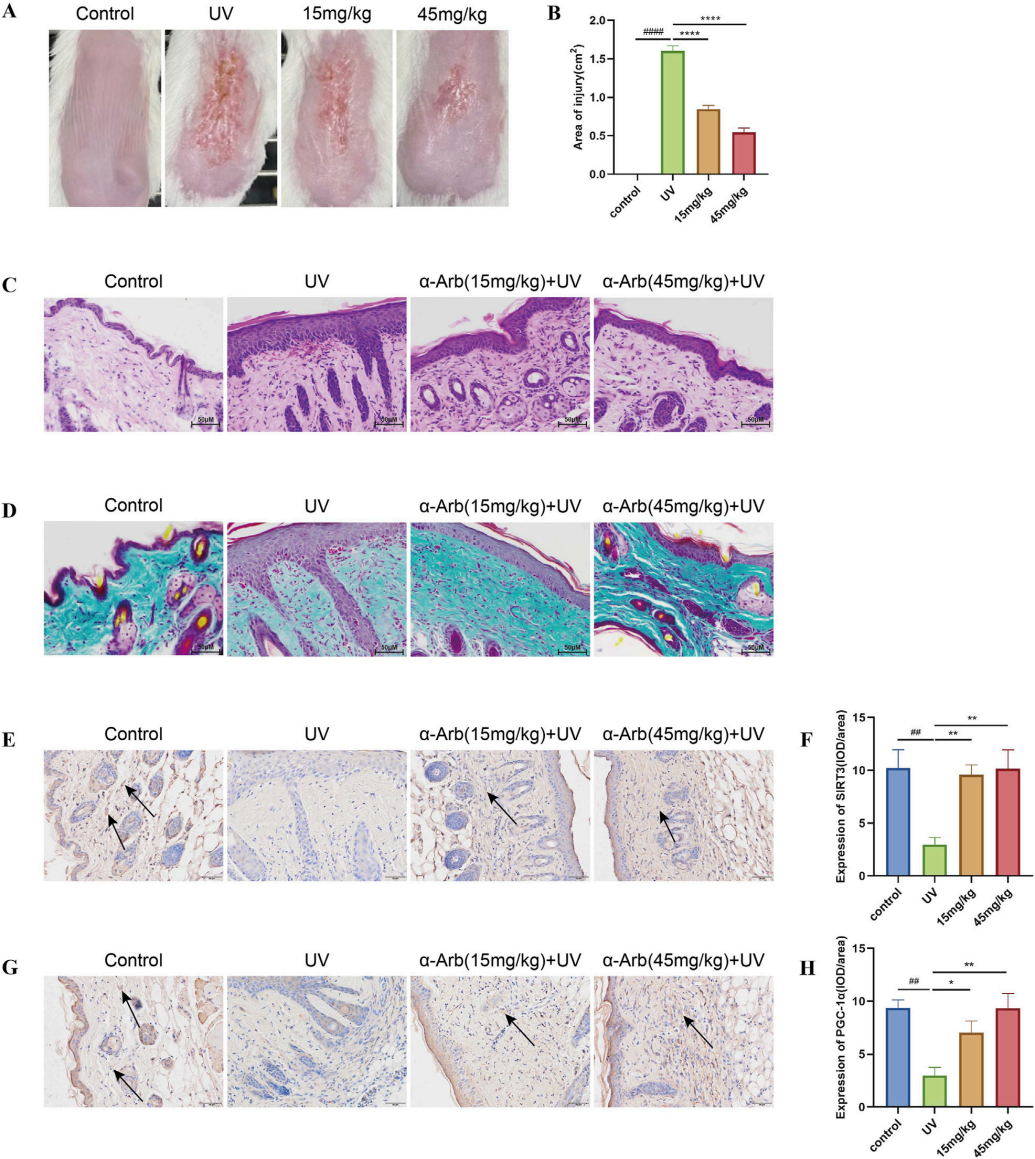
Effect of α-arbutin on the macroscopic appearance and histological changes in photoaged skin of mice. **(A)** Macroscopic changes in the dorsal skin of mice. **(B)** Area of injury. **(C)** Representative sections stained with H&E. **(D)** Masson’s trichrome staining detected loss of dermal collagen fibers in the skin of mice. Expression of SIRT3 **(E)** and PGC-1α **(G)** was investigated by immunohistochemical staining. IOD value of SIRT3 **(F)** and PGC-1α **(H)**. ####*P* < 0.0001 vs. control group, ##*P* < 0.01 vs. control group, ****P < 0.0001 vs. UV group, **P < 0.01 vs. UV group.

Throughout the experimental period, the skin of non-irradiated mice did not exhibit significant macroscopic changes except for slight age-related wrinkles. This finding indicated that shaving did not cause macroscopic damage to the skin. From day 5, the mice in the model group developed skin conditions, such as dryness, redness, and thickening. Over the 15-day experimental period, the mice treated with α-arbutin hydrogel showed partial improvement in UV-induced skin changes, with a significant reduction in the redness area (Figures 7A, B).

The histological changes in the skin of the back were analyzed using H&E and Masson’s trichrome staining to evaluate the effects of α-arbutin (Figures 7C, D). The epidermis of the skin exposed to UV radiation thickened, with inflammatory cell infiltration noted in the dermis and significant dilation of blood vessels. The collagen fibers in the dermis were sparse and disorganized. Application of a hydrogel containing α-arbutin alleviated UV-induced structural damage to the skin of mice, resulting in thinner epidermal layers, reduced infiltration of inflammatory cells in the dermis, and a well-organized structure of collagen fibers.

### 3.12 α-Arbutin increases the expression of SIRT3 and PGC-1α in photoaged skin of mice

The expression levels of SIRT3 and PGC-1α proteins in the skin of mice were detected by immunohistochemical staining. SIRT3 and PGC-1α protein expression was decreased in the skin of mice in the UVA irradiation group compared with the control group. Nevertheless, these levels were significantly increased by treatment with α-arbutin (Figures 7E, F).

## 4 Discussion

Extrinsic skin aging, also known as photoaging, refers to skin aging caused by external factors, mainly solar UV radiation. It is most frequently observed on sun-exposed sites, such as the face, lateral neck, and extensor forearms (Sachs et al., 2019). Extrinsic skin aging is characterized by deep wrinkles, leathery appearance, erythema and dryness (Hwang et al., 2018). Due to its strong inhibitory effect on tyrosinase activity, α-arbutin is widely used as a skin-lightening agent in the cosmeceutical industry (Saeedi et al., 2021; Zhu et al., 2018; Qin et al., 2014).

According to the free radical aging theory, ROS mainly derived from cellular oxidative metabolism and UV radiation, play a major role in skin aging (Gu et al., 2020; Brand et al., 2018). High levels of ROS can impair the antioxidant defense system of the skin. This results in oxidative damage to DNA mutations, lipid peroxidation, and protein oxidation, leading to cell apoptosis (Xuan et al., 2019; Dimeo et al., 2016; Schuch et al., 2017). As the second messenger of cells, ROS participates in a series of signal transduction cascades. Excessive production of ROS can result in cell damage through a variety of cell signaling pathways, such as MAPK and NF-kB, leading to oxidative stress, which causes photoaging and skin structural changes (Davinelli et al., 2018). Wang et al. (2020) determined the antioxidant capacity *in vitro* of arbutin for free radical scavenging and reducing iron. Given that the initiation and propagation of skin aging are closely related with oxidation events, it was speculated that the high-quality performance in antioxidation assay may be supplementary to the anti-photoaging capacity of α-arbutin. Consistent with these results, in the present study, we found that the levels of ROS were increased and the viability of HaCaT cells was decreased after UVA irradiation. Treatment with α-arbutin enhanced cell viability by increasing the levels of GSH and decreasing ROS generation, thereby protecting HaCaT cells from UVA-induced oxidative damage. Immunofluorescence assay of Phospho-Histone H2A.X showed that α-arbutin ameliorated UVA-induced DNA damage. Analysis through flow cytometry showed that α-arbutin reduced cell apoptosis caused by UVA radiation. These results suggest that α-arbutin protects cells from oxidative damage induced by UVA radiation. α-Arbutin is a glycosylated hydroquinone. Glycosylated natural compounds have been widely discovered in nature, like anthocyanins and gastrodin with antioxidant activity (Li et al., 2023; Zhao et al., 2023; Xiao et al., 2023). Glycosylation always renders improved physical and biological properties. β-Glucogallin is derived from gallic and possesses antioxidant, antibacterial, and extra UV-photoprotective avtivity compared with gallic acid (Shen et al., 2017; Wang et al., 2016). It speculated that the glycosylated structure confers α-arbutin antioxidant and UV protective activities.

The mitochondrial respiratory chain is the major source of intracellular ROS generation and, at the same time, an important target for the damaging effects of ROS (Gomes et al., 2020; Brillo et al., 2021). They cause associated oxidative damage to membrane structures and mitochondrial DNA, further impairing mitochondrial function and affecting cell viability, given that mitochondria and mitochondrial dysfunction play a central role in apoptosis, cancer, cellular injury, and other age-related diseases (Davinelli et al., 2018; Hseu et al., 2015). In our study, UVA-irradiated cells stained with Mito-Tracker green showed significantly less fluorescence intensity, and the mitochondrial membrane potential was also reduced. Changes in mitochondrial membrane potential could be a good indicator of impaired cellular function. However, α-arbutin ameliorated the UVA-induced decrease in mitochondrial membrane potential of HaCaT cells. Mitochondrial structure and cristae morphology determine mitochondrial respiratory efficiency (Jin et al., 2024). We observed by TEM that UVA radiation resulted in abnormal mitochondrial shape and mitochondrial cristae destruction in HaCaT cells. In contrast, pretreatment with α-arbutin effectively protected the mitochondrial structure from destruction by UVA. The mitochondrial electron transport chain is the major site of free radical production, thus the inner mitochondrial membrane (mainly at complexes I and II) is the main target of ROS damage (Gomes et al., 2020; Zou et al., 2017). In our study, UVA radiation caused a significant decrease in the activity of mitochondrial complex I/II, and the Western blot results showed a decrease in the expression levels of NDUFS1 and SDHA proteins. However, α-Arbutin pretreatment increased the activity and expression of mitochondrial complex I/II. Thus, we assume that incubation of HaCaT cells with α-arbutin prior to UVA radiation could enter and deposit in mitochondria, eliminating the ROS, and thereby ameliorating mitochondrial dysfunction.

Based on the protection of α-arbutin on mitochondrial function and integrity in UVA irradiated keratinocytes, we focused our research goal on mitochondrial biogenesis. It has been shown that SIRT3, a member of the NAD^+^-dependent deacetylase family, targets many substrates controlling mitochondrial biogenesis through lysine deacetylation and regulates mitochondrial metabolism and antioxidant mechanisms such as ROS production and clearance (Yang et al., 2016; Li and Cai, 2022; Torrens-Mas et al., 2017). In our study, molecular docking studies were performed to obtain predictions of α-arbutin and SIRT3 interactions. The results revealed that α-arbutin interacted with SIRT3 through pi-pi and hydrogen bonds. Furthermore, to evaluate whether α-arbutin could bind to SIRT3, we investigated the interaction of α-arbutin with SIRT3 using a surface plasmon response biosensor (SPR). The assay data showed the binding of α-arbutin to SIRT3 with KD (dissociation equilibrium constant) of 1.678 × 10^-4^ M, indicating an affinity of α-arbutin for SIRT3 protein. Mitochondrial biogenesis is a strictly regulated process, in which cells regulate the production of mtDNA primarily through nuclear receptors, in which PGC-1α is a key signaling molecule that regulates mitochondrial biogenesis, driving mtDNA replication and transcription (Zhang et al., 2023; Chen et al., 2022). In UVA radiation-induced HaCaT cells, we found that SIRT3 and PGC-1α protein expressions were significantly decreased, whereas α-arbutin pretreatment upregulated the expression of SIRT3 and PGC-1α proteins. Consistent with the *in vitro* experiments, the immunohistochemical analysis of mouse skin also demonstrated that α-arbutin could increase SIRT3 and PGC-1α proteins, which protected the skin from UV-induced damage. The *in vitro* and *in vivo* assays seemed to confirm the results of molecular docking and SPR, perhaps because α-arbutin was able to interact with SIRT3, so it was able to regulate SIRT3 protein expression.

The expression and activation of PGC-1α are regulated by multiple upstream pathways, such as SIRT3 overexpression leads to upregulated expression of genes and proteins (PGC-1α and TFAM) involved in mitochondrial biogenesis. Therefore, SIRT3 improves cardiac mitochondrial health and function by SIRT3-AMPK-PGC-1α axis (Li et al., 2021). However, the exact mechanism of SIRT3-mediated regulation of PGC-1α remains to be further explored. It has been reported that PGC-1α stimulates SIRT3 gene expression in mouse cells and hepatocytes (Li and Cai, 2022). However, there is also evidence showing that PGC-1α may be an important component of SIRT3-mediated molecular signaling, and the knockdown of SIRT3 results in downregulation of PGC-1α (Zeng et al., 2019). We tested whether the siRNA SIRT3 alters the expression of PGC-1α. Our results demonstrated that the siRNA knockdown of SIRT3 affect the protein expression of PGC-1α. Moreover, the siRNA knockdown of SIRT3 reduced the mRNA levels of NRF2, TFAM and D-loop expression, which α-arbutin could partially reverse. Importantly, PGC-1α interacts with two key downstream transcription factors, NRF1 and NRF2, to activate TFAM, which subsequently binds to five complex subunits in the mitochondria, thus promoting mtDNA replication and transcription (Kozhukhar and Alexeyev, 2019). These findings provide strong evidence for our study, α-arbutin markedly improved the expression of SIRT3 and PGC-1α, the mRNA transcription of NRF1 and NRF2 increased with the addition of PGC-1α protein, which leads to a decrease in intracellular ROS levels. These effects resulted in the reduction of intracellular ROS levels. Moreover, the elevated transcription level of mitochondrial TFAM could promote the production of mitochondria, thereby increasing the mitochondrial membrane potential. The results showed that the effect of α-arbutin on UVA-induced photoaging in HaCaT cells was mainly mediated through SIRT3/PGC-1α activation.

## 5 Conclusion

**FIGURE 8:**
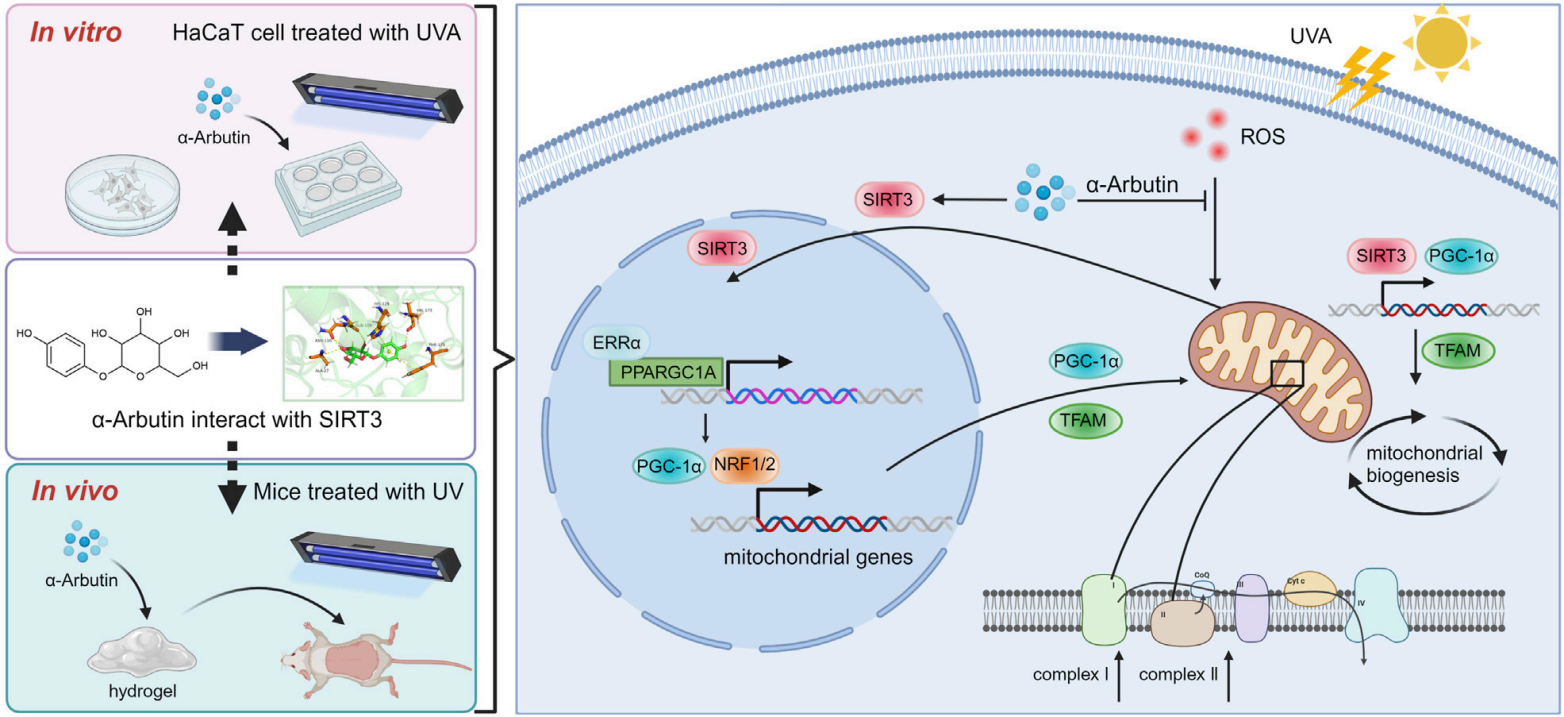
Mechanism of α-arbutin against UVA-induced photoaging (Created by Biorender.com).

In our study, the protective effects of α-arbutin and the underlying biological mechanisms against UVA-induced photoaging were investigated as shown in Figure 8. Treatment with α-arbutin significantly augmented PGC-1α expression by upregulating SIRT3. In addition, the intracellular ROS content decreased with the elevated levels of PGC-1α expression and NRF expression. Additionally, the increased level of TFAM transcription promoted mitochondrial production. In UV-irradiated mouse dorsal skin after application of α-arbutin hydrogel, we observed an increase in the expression of SIRT3 and PGC-1α proteins, and the level of collagen. In this study, we demonstrated that the protective effect of α-arbutin on UVA-induced photoaging in HaCaT cells and mice was mainly mediated by activating SIRT3/PGC-1α. But it is unclear whether α-arbutin can improve photoaging by modulating other factors, and further research is needed. In conclusion, the results suggest that α-arbutin could be added into skin care products for protection skin from UV damage.

## Data Availability

The raw data supporting the conclusions of this article will be made available by the authors, without undue reservation.

## Abbreviations

ETC, electron transport chain; LDH, Lactate dehydrogenase; MMP, Mitochondrial membrane potential; PGC-1α, peroxisome proliferator-activated receptor coactivator 1α; SIRT3, Sirtuin3; TFAM, mitochondrial transcription factor A.
